# Dendritic-Cell-Vaccine-Based Immunotherapy for Hepatocellular Carcinoma: Clinical Trials and Recent Preclinical Studies

**DOI:** 10.3390/cancers14184380

**Published:** 2022-09-08

**Authors:** Long-Bin Jeng, Li-Ying Liao, Fu-Ying Shih, Chiao-Fang Teng

**Affiliations:** 1 Organ Transplantation Center, China Medical University Hospital, Taichung 404, Taiwan; 2 Cell Therapy Center, China Medical University Hospital, Taichung 404, Taiwan; 3 Development of Plastic and Reconstructive Surgery, China Medical University Hospital, Taichung 404, Taiwan; 4 Ph.D. Program for Biotech Pharmaceutical Industry, School of Pharmacy, China Medical University, Taichung 404, Taiwan; 5 Graduate Institute of Biomedical Sciences, China Medical University, Taichung 404, Taiwan; 6 Program for Cancer Biology and Drug Development, China Medical University, Taichung 404, Taiwan; 7 Research Center for Cancer Biology, China Medical University, Taichung 404, Taiwan

**Keywords:** hepatocellular carcinoma, dendritic-cell vaccine, immunotherapy, clinical trials, preclinical studies

## Abstract

**Simple Summary:**

This review summarizes the evidence from clinical trials and recent preclinical studies regarding the evaluation and optimization of dendritic cells (DCs)-based vaccines as either mono- or combination immunotherapy with current anticancer therapies and/or various immune effector cells for treating hepatocellular carcinoma (HCC).

**Abstract:**

Although many surgical and nonsurgical therapeutic options have been well-established, hepatocellular carcinoma (HCC) remains the third most common cause of cancer-related death worldwide. Therefore, the discovery of novel potential therapeutic strategies is still urgently required for improving survival and prognosis of HCC patients. As the most potent antigen-presenting cells in the human immune system, dendritic cells (DCs) play an important role in activating not only innate but also adaptive immune responses to specifically destroy tumor cells. As a result, DC-based vaccines, which are prepared by different tumor-antigen-pulsing strategies or maturation-stimulating reagents, either alone or in combination with various anticancer therapies and/or immune effector cells, have been developed as a promising personalized cancer immunotherapy. This review provides a comprehensive summary of the evidence from clinical trials evaluating the safety, feasibility, and efficacy of DC-based vaccines in treating HCC patients and highlights the data from recent preclinical studies regarding the development of promising strategies for optimizing the efficacy of DC-vaccine-based immunotherapy for HCC.

## 1. Introduction

Hepatocellular carcinoma (HCC) accounts for up to 90% of primary liver cancers and ranks as the sixth most prevalent human cancer worldwide [1,2]. Many surgical, locoregional, and systemic therapeutic options have been well-established for treating HCC, such as liver transplantation, surgical resection, radiofrequency ablation (RFA), percutaneous microwave ablation (PMWA), percutaneous ethanol injection (PEI), transarterial embolization/chemoembolization (TAE/TACE), external beam radiation therapy (EBRT), chemotherapy, and molecular-targeted therapy [3,4,5]. Moreover, both sorafenib and lenvatinib have been approved by the US Food and Drug Administration (FDA) as molecular-targeted drugs for the first-line treatment of advanced primary HCC [6,7]. However, the overall prognosis of HCC patients remains dismal, rendering HCC as the third leading cause of cancer-related death worldwide and resulting in over 800,000 deaths every year [8,9]. Therefore, the development of new and effective therapeutic strategies for HCC is an important goal to improve patient survival.

Dendritic cells (DCs) are the most potent antigen-presenting cells in the human immune system and play a crucial role in not only the activation of innate immunity but also the induction of cytotoxic T lymphocyte (CTL)-mediated adaptive immunity [10,11,12]. In their immature state, DCs circulate broadly in the blood and peripheral tissues, where they sample antigens that are derived from pathogen-infected or tumor cells. After the uptake of presentable antigens, DCs undergo a process of phenotypical and functional maturation and migrate to the secondary lymphoid tissues, such as lymph nodes, where they present processed antigens to and activate CTLs, which thereby trigger an antigen-specific immune response to eliminate antigen-expressing target cells. Additionally, mature DCs are also capable of enhancing the cytotoxic activity of nature killer cells (NKs), which function as innate immune effector cells to destroy pathogen-infected or tumor cells [13,14]. Based on these characteristics, DC-based vaccines have emerged as a promising immunotherapy for treating many types of cancer, including HCC [15,16,17].

In this review, we summarize the available results from clinical trials that have been conducted so far to evaluate the safety, feasibility, and efficacy of DC-based vaccines, either alone or in combination with various anticancer therapies and/or immune effector cells, in treating HCC patients (Table 1 and Table 2). Moreover, we highlight the promising data from recent in vitro and in vivo preclinical studies since 2019 regarding DC-vaccine-based immunotherapy for HCC (Table 3).

## 2. Monotherapy of Whole-Tumor-Cell-Lysate-Pulsed or Specific-Tumor-Antigen-Pulsed DC Vaccines for Treating HCC Patients

Four major strategies have been applied to pulse DCs with tumor antigens in vitro for the preparation of antitumor DC vaccines: the first one involves the co-culture of DCs with frozen–thawed total lysates of autologous tumors or allogeneic tumor cell lines [18,19]; the second involves the co-culture of DCs with synthetic peptides or recombinant proteins of known tumor antigens [20,21]; the third one involves the transfection of DCs with plasmid DNA or viral vector DNA or messenger RNA (mRNA) that contains the genes encoding known tumor antigens [22,23]; and fourth one involves the fusion of DCs with entire tumor cells through the mediation of polyethylene glycol, a widely used fusion agent of lipid membranes [24]. Except for the fourth strategy, which is still under preclinical evaluation (mentioned later in Section 4), the clinical results of monotherapy based on DC vaccines which are prepared by the other three antigen-pulsing strategies for HCC patients have been evaluated in several completed and ongoing clinical trials (Table 1).

**Table 1:** Summary of clinical trials regarding DC-vaccine-based mono-immunotherapy for treating HCC patients.

Treatment	Disease Stage Targeted	Clinical Trial Identifier	Start Year	Patient Number	Phase	Status	Clinical Results	Publication
Autologous-HCC-tumor-lysate-pulsed mature-DC vaccine	Unresectable primary HCC	NA	2000	8	I	Completed	Safe and well tolerated 50% of patients with SD One patient with decreased tumor size and another with decreased serum levels of AFP and PIVKA-II	Iwashita et al. [25]
Autologous-HCC-tumor-lysate-pulsed mature-DC vaccine	Advanced primary HCC, AJCC TNM (5th edition) stage IVA and IVB	NA	2000	31	NA	Completed	Safe and well tolerated 12.9% of patients with PR and 54.8% with SD; 1-year OS rate of 40.1%; better 1-year OS rate in patients with initial and boost vaccinations than patients with initial vaccination alone (63.3% versus 10.7%)	Lee et al. [26]
Autologous-HCC-tumor-lysate-pulsed mature-DC vaccine	Primary HCC	NCT00327496	2006	10	NA	Completed	NA	NA
HepG2-HCC-cell-lysate-pulsed mature-DC vaccine	Advanced primary HCC	NA	NA	35	II	Completed	Safe and well tolerated 4% of patients with PR and 24% with SD; 6-month and 1-year OS rates of 33% and 11%, respectively 23.5% of patients with decreased serum levels of AFP	Palmer et al. [27]
HepG2-HCC-cell-lysate-pulsed mature-DC vaccine	Advanced primary HCC, Child–Pugh class B or C	NA	2009	30	NA	Completed	Safe and well tolerated 13.3% with PR and 60% with SD in patients with vaccination compared to none with PR and 13.3% with SD in patients with supportive treatment; longer median OS time for patients with vaccination than patients with supportive treatment (7 months versus 4 months)	EI Ansary et al. [28]
Mature-DC vaccine co-pulsed with four AFP peptides: AFP_137–145_, AFP_158–166_, AFP_325–334_, and AFP_542–550_	Primary HCC, AJCC TNM (5th edition) stage IIIA to IVB, Child–Pugh class A or B, class I MHC molecule HLA-A*0201 positive, AFP positive	NCT00022334	2003	10	I/II	Completed	Safe and well tolerated 60% of patients with increased IFN-γ-producing AFP-specific CTL responses	Butterfield et al. [29]
HSP70-mRNA-transfected mature-DC vaccine	Unresectable HCV-related primary or recurrent HCC	NA	2007	12	I	Completed	Safe and well tolerated 16.7% of patients with CR and 41.7% with SD One evaluable patient with increased tumor-infiltrating granzyme B-expressing CTLs	Maeda et al. [30]
Mature-DC vaccine co-pulsed with AFP, MAGE-1, and GPC-3 proteins	Refractory primary or recurrent HCC	JPRN-UMIN000011854	2013	5	I	Completed	NA	NA
Personalized HCC-tumor-antigen-pulsed mature-DC vaccine	Primary HCC, BCLC stage B or C, Child–Pugh class A or B	ChiCTR1900021177	2018	30	NA	Ongoing	NA	NA

Abbreviations: DC, dendritic cell; HCC, hepatocellular carcinoma; NA, not available; SD, stable disease; AFP, alpha-fetoprotein; PIVKA-II, protein induced by vitamin K absence or antagonist II; AJCC, American Joint Committee on Cancer; TNM, tumor–node–metastasis; PR, partial response; OS, overall survival; MHC, major histocompatibility complex; HLA, human leukocyte antigen; IFN-γ, interferon-gamma; CTL, cytotoxic T lymphocyte; HSP70, heat-shock protein 70; CR, complete response; mRNA, messenger RNA; HCV, hepatitis C virus; MAGE-1, melanoma-associated antigen 1; GPC-3, glypican-3; BCLC, Barcelona Clinic Liver Cancer.

### 2.1. Autologous Tumor-Lysate-Pulsed DC Vaccines

A completed phase I clinical trial conducted by Iwashita et al. evaluated the safety and feasibility of a mature-DC vaccine, which was pulsed with total lysate prepared from autologous HCC tumors of patients, in treating eight patients with unresectable primary HCC [25]. All the patients were vaccinated with 1 × 10^6^ to 1 × 10^7^ of DCs intranodally four times at 1-week intervals. After the initial four vaccinations, half of the patients were further vaccinated at monthly intervals for up to 12 vaccinations. The results showed that DC vaccination was safe and well tolerated in all patients. Among all patients, half exhibited stable disease (SD), one had decreased tumor size (from 13 mm to 7 mm in diameter), and another had lowered serum levels of HCC tumor markers such as alpha-fetoprotein (AFP) and protein induced by vitamin K absence or antagonist II (PIVKA-II) after vaccination. Another completed clinical trial conducted by Lee et al. further evaluated the safety and feasibility of an autologous-HCC-tumor-lysate-pulsed mature-DC vaccine in treating 31 patients with advanced primary HCC [26]. All the patients received an initial five vaccinations of DCs with a median of 2.5 × 10^6^ cells each time intravenously at 1-week intervals. Seventeen of the patients further received boost vaccinations at monthly intervals for 2 to 12 months. The results showed that DC vaccination was safe and well tolerated in all patients. Among all patients, 4 (12.9%) displayed a partial response (PR), and 17 (54.8%) achieved SD; the 1-year overall survival (OS) rate was 40.1%. The patients who received initial and boost vaccinations had a significantly better 1-year OS rate than the patients who received initial vaccination alone (63.3% versus 10.7%, *p*-value < 0.001). Another completed clinical trial (NCT00327496) evaluated the efficacy of an autologous HCC-tumor-lysate-pulsed mature-DC vaccine in stimulation of CTL-mediated cytotoxicity against primarily cultured HCC cells; however, the results are not available. Collectively, these clinical trials support that autologous-tumor-lysate-pulsed DC vaccines are safe and feasible as monotherapy for treating HCC patients who are not eligible for current therapeutic methods; however, the efficacy needs to be evaluated in further studies.

### 2.2. Allogeneic-Tumor-Cell-Line-Lysate-Pulsed DC Vaccines

A completed phase II clinical trial conducted by Palmer et al. evaluated the safety and efficacy of a mature-DC vaccine, which was pulsed with total cell lysate prepared from human HCC cell line HepG2, in treating 35 patients with advanced primary HCC [27]. All the patients received a maximum of six DC vaccinations intravenously at 3-week intervals. The results showed that DC vaccination was safe and well tolerated in all patients. Among 25 patients who received at least three vaccinations, 1 (4%) developed a PR, and 6 (24%) achieved SD. Among 17 patients who had a baseline serum level of AFP ≥ 1000 ng/mL, 4 (23.5%) had decreased AFP levels (<30% of baseline) after vaccination. The 6-month and 1-year OS rates were 33% and 11%, respectively. Another completed clinical trial conducted by EI Ansary et al. also evaluated the safety and efficacy of a HepG2-cell-lysate-pulsed mature-DC vaccine in treating patients with advanced primary HCC [28]. A total of 30 patients were divided into two groups with no difference in baseline characteristics: in group I, 15 patients received intradermal DC vaccination with a total of 2 × 10^7^ cells; and in group II, 15 patients received supportive treatment as a control group. The results showed that DC vaccination was safe and well tolerated in all patients. After a 6-month follow-up period, in group I of patients, two (13.3%) achieved a PR, and nine (60%) had SD; in contrast, in group II, none achieved a PR, and only two (13.3%) had SD. The group I of patients exhibited a significantly longer median OS time than the group II of patients (7 months versus 4 months, *p*-value = 0.008). Taken together, these clinical trials indicate that monotherapy based on allogeneic-tumor-cell-line-lysate-pulsed DC vaccines is a safe and effective therapeutic option for HCC patients who cannot be treated with current treatment approaches; however, the efficacy still needs to be further improved.

### 2.3. Specific-Tumor-Antigen-Pulsed DC Vaccines

A completed phase I/II clinical trial (NCT00022334) conducted by Butterfield et al. evaluated the safety and feasibility of a mature-DC vaccine, which was co-pulsed with four immunodominant AFP-derived peptides (AFP_137_**_–_**_145_, AFP_158–166_, AFP_325–334_, and AFP_542–550_), in treating 10 patients with primary HCC who were positive for class I major histocompatibility complex (MHC) molecule human leukocyte antigen (HLA)-A*0201 and AFP [29]. All the patients were vaccinated with 1 × 10^6^ to 1 × 10^7^ of DCs intradermally three times at 2-week intervals. The results showed that DC vaccination was safe and well tolerated in all patients. Six of the patients (60%) exhibited increased interferon-gamma (IFN-γ)-producing AFP-specific CTL responses after vaccination. Another completed phase I clinical trial conducted by Maeda et al. evaluated the safety and feasibility of a mature-DC vaccine, which was transfected with mRNA of the HCC tumor marker heat-shock protein 70 (HSP70), in treating 12 patients with unresectable hepatitis C virus (HCV)-related primary or recurrent HCC [30]. All the patients were vaccinated with 1 × 10^7^ to 3 × 10^7^ of DCs intradermally three times at 3-week intervals. The results showed that DC vaccination was safe and well tolerated in all patients. Among all patients, two (16.7%) achieved a complete response (CR), and five (41.7%) had SD. Tumor specimens were obtained from one patient and revealed increased infiltration of granzyme B-expressing CTLs after vaccination. Another completed phase I clinical trial (JPRN-UMIN000011854) evaluated the safety and feasibility of a mature-DC vaccine, which was co-pulsed with the recombinant proteins of three HCC tumor markers, namely AFP, melanoma-associated antigen 1 (MAGE-1), and glypican-3 (GPC-3), in treating patients with refractory primary or recurrent HCC. Another ongoing clinical trial (ChiCTR1900021177) was aimed at evaluating the clinical results of a personalized tumor-antigen-pulsed DC vaccine in treating patients with primary HCC. However, there are no available results from the two trials mentioned. Overall, these clinical trials suggest that specific-tumor-antigen-pulsed DC vaccines as monotherapy is safe and feasible in treating HCC patients who are not appropriate candidates for current therapeutic approaches; however, further studies are still needed to evaluate and optimize the efficacy.

## 3. Combination Therapy of DC-Based Vaccines and Anticancer Therapies or Immune Effector Cells for Treating HCC Patients

Although many surgical and nonsurgical anticancer therapies have been developed for treating HCC, the therapeutic benefits of each therapy alone on the prognosis and survival of patients remain limited due to either tumor recurrence or drug resistance [3,4,5]. Additionally, tumor cells evolve multiple mechanisms to escape immune attack, such as the expression of immune checkpoint molecules to suppress the antitumor activity of CTLs [31,32]. As a result, monoclonal antibodies, which block the interaction between immune checkpoint molecules, also known as immune checkpoint inhibitors (ICIs), have emerged as potential immunotherapeutic agents for cancer [33,34]. Especially, nivolumab, an ICI that targets programmed death 1 (PD-1), has been approved by the US FDA as a second-line treatment for advanced primary HCC [35]. However, similar to patients with other solid tumors, HCC patients display a low response rate to ICI monotherapy [36,37]. Therefore, the combination of DC vaccines and various anticancer therapies has been derived, and the clinical results of this combination therapy for HCC patients have been evaluated in many completed and ongoing clinical trials (Table 2). Moreover, based on the capability of DCs to activate and enhance the tumor-killing activity of CTLs and NKs [38], the combination of DC vaccines and such immune effector cells or the infusion of DC-activated immune effector cells has been developed and evaluated in treating HCC patients in several clinical trials (Table 2).

**Table 2:** Summary of clinical trials regarding DC-vaccine-based combination immunotherapy for treating HCC patients.

Treatment	Disease Stage Targeted	Clinical Trial Identifier	Start Year	Patient Number	Phase	Status	Clinical Results	Publication
**Combination of DC Vaccines and Anticancer Therapies**								
Immature-DC vaccine combined with EBRT	Advanced primary HCC	NA	2001	12	I	Completed	Safe and well tolerated 16.7% of patients with PR 25% of patients with decreased serum levels of AFP	Chi et al. [39]
Mature-DC vaccine combined with EBRT	Unresectable primary HCC, AJCC TNM (8th edition) stage IIIA to IVB, Child–Pugh class A	NCT03942328	2019	26	Early I	Ongoing	NA	NA
Mature-DC vaccine combined with RFA	HCV-related primary HCC	JPRN-C000000451	2006	5	NA	Completed	NA	NA
OK432-stimulated mature-DC vaccine combined with RFA	HCV-related primary HCC, Child–Pugh class A or B	JPRN-UMIN000001701	2009	30	I/II	Completed	Safe and well tolerated Longer median RFS time for patients with OK432-stimulated DC vaccination than patients with non-OK432-stimulated DC vaccination (24.8 months versus 13.0 months)	Kitahara et al. [40]
Mature-DC vaccine combined with TAE	Primary HCC, Child–Pugh class A or B	JPRN-UMIN000012702	2013	3	NA	Completed	NA	NA
Mature-DC vaccine combined with TAE and RFA	Unresectable primary HCC, Child–Pugh class A or B	JPRN-UMIN000036065	2019	3	I	Completed	NA	NA
Mature-DC vaccine combined with TAE and RFA	Unresectable primary HCC, Child–Pugh class A or B	JPRN-jRCTc050200107	2021	30	I/II	Ongoing	NA	NA
Mature-DC vaccine (ilixadencel) co-activated with TLR3 and 7/8 agonists and IFN-γ and given in combination with molecular-targeted drug sorafenib	Advanced primary HCC, BCLC stage B or C, Child–Pugh class A	NCT01974661	2013	17	I	Completed	Safe and well tolerated 5.9% of patients with PR and 29.4% with SD; median TTP and OS times of 5.5 months and 7.5 months, respectively 73.3% of evaluable patients with increased frequency of IFN-γ-producing CTLs in peripheral blood	Rizell et al. [41]
Autologous-irradiated-HCC-tumor-stem-cell-pulsed mature-DC vaccine combined with surgical resection and TACE	Unresectable HBV-related primary HCC, BCLC stage A or C, Child–Pugh class A	NA	2013	8	I	Completed	Safe and well tolerated No increase in serum levels of hepatic transaminases, hepatitis B antigens, and viral DNA	Wang et al. [42]
HepG2-HCC-cell-lysate-pulsed mature-DC vaccine combined with TACE	Primary HCC, Child–Pugh class A or B	ISRCTN11889464	2014	48	II	Completed	NA	NA
HepG2-HCC-cell-lysate-pulsed mature-DC vaccine combined with TACE	HCV-related primary HCC, BCLC stage B or D, Child–Pugh class A or B or C	DRKS00016606	2015	20	II	Completed	Safe and well tolerated 60% with PR and 20% with SD in BCLC stage B patients with combined therapy similar to patients with TACE alone; 20% with PR and 40% with SD in BCLC stage D patients with vaccination compared to none with PR or SD in patients with supportive treatment Increased frequency of peripheral CTLs and decreased serum levels of AFP	Abdel Ghafar et al. [43]
Mature-DC vaccine co-pulsed with HBV-specific antigen peptides and HepG2 HCC cell lysate and given in combination with TACE	Unresectable HBV-related primary HCC, BCLC stage B or C, Child–Pugh class A or B	NCT03086564	2017	70	I/II	Completed	NA	NA
Peptides-pulsed mature-DC vaccine combined with RFA	Primary HCC, HLA-A24 positive	JPRN-UMIN000020811	2016	10	NA	Completed	NA	NA
Peptides-pulsed mature-DC vaccine combined with RFA	Primary HCC, Child–Pugh class A or B	JPRN-jRCTc040190093	2020	6	I	Ongoing	NA	NA
HSP70-mRNA-transfected mature-DC vaccine combined with surgical resection	Resectable primary HCC, LCSGJ (5th edition) stage II to IVA	JPRN-UMIN000010691	2012	45	I/II	Completed	Safe and well tolerated Better median OS and DFS times for patients with HSP70-expressing HCC with combined therapy than patients with surgical resection alone	Matsui et al. [44]
Mature-DC vaccine co-pulsed with AFP, MAGE-1, and GPC-3 proteins and given in combination with TACE	Primary HCC, AJCC TNM (6th edition) stage II to IIIC, Child–Pugh class A or B	NA	2009	5	I/II	Completed	Safe and well tolerated 20% of patients with SD 100% of patients with increased IFN-γ-producing CTL responses against AFP, MAGE-1, and/or GPC-3 antigens	Tada et al. [45]
Mature-DC vaccine co-pulsed with AFP, MAGE-1, and GPC-3 proteins and given in combination with TACE	Unresectable primary HCC, Child–Pugh class A	KCT0000986	2013	40	II	Ongoing	NA	NA
Mature-DC vaccine co-pulsed with AFP, MAGE-1, and GPC-3 proteins and given in combination with surgical resection	Primary HCC	JPRN-UMIN000021545	2016	50	II	Completed	NA	NA
Mature-DC vaccine co-pulsed with AFP, MAGE-1, and GPC-3 proteins and given in combination with surgical resection, RFA, PEI, or TACE	Primary HCC, AJCC TNM (6th edition) stage I to IIIC, Child–Pugh class A or B	KCT0000427	2009	12	I/IIa	Completed	Safe and well tolerated 75% of patients free of tumor recurrence up to 24 weeks and with stronger IFN-γ-producing CTL responses against AFP, MAGE-1, and/or GPC-3 antigens Longer median TTP for patients with vaccination than patients without vaccination (36.6 months versus 11.8 moths)	Lee et al. [46]
Mature-DC vaccine co-pulsed with AFP, MAGE-1, and GPC-3 proteins and given in combination with surgical resection, RFA, PEI, or TACE	Primary HCC, AJCC TNM (6th edition) stage I to IIIC, Child–Pugh class A or B	KCT0000008	2010	156	II	Completed	Safe and well tolerated 63% of patients with increased IFN-γ-producing CTL responses against AFP, MAGE-1, and/or GPC-3 antigens Better RFS for non-RFA-treated patients with vaccination than patients without vaccination	Lee et al. [47]
HCC-tumor-neoantigen-pulsed mature-DC vaccine combined with PMWA	Primary HCC, HKLC stage IIa, Child–Pugh class A or B	NCT03674073	2018	24	I	Ongoing	NA	NA
HCC-tumor-neoantigen-pulsed mature-DC vaccine combined with ICI nivolumab and surgical resection	Resectable primary or recurrent HCC, Child–Pugh class A	NCT04912765	2021	60	II	Ongoing	NA	NA
Multiple-HCC-tumor-antigens-pulsed mature-DC vaccine combined with surgical resection, TACE, or molecular-targeted drug sorafenib or lenvatinib	HBV-related primary HCC, Child–Pugh class A or B	NCT04317248	2020	600	II	Ongoing	NA	NA
**Combination of DC vaccines and immune effector cells and anticancer therapies**								
Autologous-HCC-tumor-lysate-pulsed mature-DC vaccine with CATs and combined with surgical resection	Primary HCC	NA	2000	94	II	Completed	Safe and well tolerated Longer median OS (97.7 months versus 41.0 months) and RFS (24.5 months versus 12.6 months) times for patients with combined therapy than patients with surgical resection alone	Shimizu et al. [48]
Autologous-HCC-tumor-lysate-pulsed mature-DC vaccine with immature DCs, CIKs, mature-DC-precision CTLs, and mature DC-CIKs combined with PMWA	HBV-related primary HCC, Child–Pugh class A or B	NA	NA	10	I	Completed	Safe and well tolerated 57.1% and 28.6% of evaluable patients with decreased and undetectable serum levels of viral DNA, respectively	Zhou et al. [49]
Mature DC-CIKs combined with surgical resection or TACE	Primary HCC	NCT01821482	2013	100	II	Ongoing	NA	NA
Mature-DC vaccine with CIKs and combined with molecular-targeted drug sorafenib	Advanced primary HCC, BCLC stage B or C, Child–Pugh class A or B	NA	2015	71	NA	Completed	Safe and well tolerated 11.4% with CR, 40% with PR, and 37.1% with SD in patients with combined therapy compared to 2.8% with CR, 13.9% with PR, and 25% with SD in patients with sorafenib alone; longer median OS time for patients with combined therapy than patients with sorafenib alone (18.6 months versus 13.8 moths) Decreased serum levels of AFP	Zhou et al. [50]
Mature-DC-precision multiple-antigen CTLs combined with surgical resection	Primary HCC, Child–Pugh class A or B	NCT02632188	2015	60	I/II	Ongoing	NA	NA
Mature-DC-precision multiple-antigen CTLs combined with TACE	Unresectable primary or recurrent HCC, Child–Pugh class A or B	NCT02638857	2015	60	I/II	Ongoing	NA	NA
Personalized HCC-tumor-neoantigen-pulsed mature-DC vaccine with mature-DC-precision neoantigen CTLs and combined with surgical resection or RFA	Primary HCC, Child–Pugh class A or B	NCT03067493	2017	10	II	Completed	Safe and well tolerated 50% of patients free of tumor recurrence for 2 years; 70% of patients with generation of de novo multiclonal neoantigen-specific CTL responses Median DFS time of 18.3 months; better median DFS time for patients who generated immune responses than patients who did not generate immune responses	Peng et al. [51]

Abbreviations: DC, dendritic cell; HCC, hepatocellular carcinoma; EBRT, external beam radiation therapy; NA, not available; PR, partial response; AFP, alpha-fetoprotein; AJCC, American Joint Committee on Cancer; TNM, tumor–node–metastasis; RFA, radiofrequency ablation; HCV, hepatitis C virus; TAE, transarterial embolization; TLR, Toll-like receptor; IFN-γ, interferon-gamma; BCLC, Barcelona Clinic Liver Cancer; SD, stable disease; TTP, time to progression; OS, overall survival; CTL, cytotoxic T lymphocyte; TACE, transarterial chemoembolization; HBV, hepatitis B virus; HLA, human leukocyte antigen; HSP70, heat-shock protein 70; LCSGJ, Liver Cancer Study Group of Japan; DFS, disease-free survival; MAGE-1, melanoma-associated antigen 1; GPC-3, glypican-3; PEI, percutaneous ethanol injection; PMWA, percutaneous microwave ablation; HKLC, Hong Kong Liver Cancer; ICI, immune checkpoint inhibitor; CAT, CD3-activated T cell; CIK, cytokine-induced killer cell; DC-CIK, dendritic-cell-activated cytokine-induced killer cell; CR, complete response.

### 3.1. Non-Antigen-Pulsed DC Vaccines Combined with Anticancer Therapies

A completed phase I clinical trial conducted by Chi et al. evaluated the safety and feasibility of an immature-DC vaccine combined with EBRT, a type of radiotherapy that deliveries radiation beams from outside the body toward tumors inside the body, in treating 12 patients with advanced primary HCC [39]. All the patients were vaccinated with 5 × 10^6^ to 5 × 10^7^ of DCs intratumorally two times at 3-week intervals 2 days after EBRT. The results showed that DC vaccination was safe and well tolerated in all patients. Among all patients, two (16.7%) achieved a PR, and three (25%) had decreased serum levels of AFP (<50% of baseline) after vaccination. Another ongoing early phase I clinical trial (NCT03942328) was aimed at evaluating the clinical results of a mature-DC vaccine combined with EBRT in treating patients with unresectable primary HCC. Another completed clinical trial (JPRN-C000000451) evaluated the clinical results of a mature-DC vaccine combined with RFA in treating patients with HCV-related primary HCC. However, there are no available results from the two trials mentioned. Another completed phase I/II clinical trial (JPRN-UMIN000001701) conducted by Kitahara et al. further evaluated the safety and efficacy of a mature-DC vaccine, which was stimulated with the Streptococcus-derived anticancer immunotherapeutic agent OK432 as a maturation reagent, combined with RFA in treating patients with HCV-related primary HCC [40]. A total of 30 patients were divided into two groups, with no difference in baseline characteristics: in group I, 16 patients were vaccinated with 5 × 10^6^ of OK432-stimulated DCs percutaneously after RFA; and in group II, 14 patients w were vaccinated with 5 × 10^6^ of non-OK432-stimulated DCs percutaneously after RFA as a control group. The results showed that DC vaccination was safe and well tolerated in all patients. Although there was no difference in OS between the two groups of patients, the median RFS time was significantly longer in group I than in group II (24.8 months versus 13.0 months, *p*-value = 0.003). Another completed clinical trial (JPRN-UMIN000012702) evaluated the clinical results of a mature-DC vaccine combined with TAE in treating patients with primary HCC. In another completed phase I (JPRN-UMIN000036065) trial and an ongoing phase I/II (JPRN-jRCTc050200107) clinical trial, the clinical results of a mature-DC vaccine combined with TAE and RFA in treating patients with unresectable primary HCC were evaluated. However, there are no available results from the three trials mentioned. Another completed phase I clinical trial (NCT01974661) conducted by Rizell et al. evaluated the safety and feasibility of a mature-DC vaccine (also known as ilixadencel) co-activated with Toll-like receptor (TLR) 3 and 7/8 agonists and IFN-γ, either alone or combined with the molecular-targeted drug sorafenib, in treating 17 patients with advanced primary HCC [41]. All the patients received three intratumoral DC vaccinations at 2-to-5-week intervals with a dose of 1 × 10^7^ cells alone (six patients; group I) or combined with sorafenib (six patients; group II) or with a dose of 2 × 10^7^ cells alone (five patients; group III). The results showed that DC vaccination was safe and well tolerated in all patients. Among all patients, one (5.9%; from group III) exhibited a PR, and five (29.4%; three from group I, one from group II, and one from group III) had SD; the median time to progression (TTP) was 5.5 months, and the median OS time was 7.5 months. A total of 11 of the 15 evaluable patients (73.3%; 9 of 11 evaluable patients from group I and group III and 2 of 4 evaluable patients from group II) displayed an elevated frequency of tumor-specific IFN-γ-producing CTLs in peripheral blood after vaccination. Collectively, these clinical trials indicate that non-antigen-pulsed DC vaccines are safe and feasible as adjuvant therapy for treating HCC patients who receive standard anticancer therapies; however, the efficacy needs to be further evaluated.

### 3.2. Autologous-Tumor-Lysate-Pulsed, Allogeneic-Tumor-Cell-Line-Lysate-Pulsed, or Specific-Tumor-Antigen-Pulsed DC Vaccines Combined with Anticancer Therapies

A completed phase I clinical trial conducted by Wang et al. evaluated the safety of an autologous-irradiated-HCC-tumor-stem-cell-pulsed mature-DC vaccine combined with surgical resection and TACE in treating eight patients with unresectable hepatitis B virus (HBV)-related primary HCC [42]. All the patients were vaccinated with 3 × 10^6^ to 2 × 10^7^ of DCs subcutaneously three times at 1-week intervals 6 weeks after TACE following surgical resection. The results showed that DC vaccination was safe and well tolerated in all patients. There was no increase in the serum levels of hepatic transaminases, hepatitis B antigens, and viral DNA after vaccination. Another completed phase II clinical trial (ISRCTN11889464) evaluated the clinical results of a HepG2-cell-lysate-pulsed mature-DC vaccine combined with TACE in treating patients with primary HCC, though the results are not available. Another completed phase II clinical trial (DRKS00016606) conducted by Abdel Ghafar et al. further evaluated the safety and efficacy of a HepG2-cell-lysate-pulsed mature-DC vaccine, either alone or combined with TACE, in treating patients with primary HCC [43]. A total of 20 patients were divided into four groups with no difference in baseline characteristics: in group I, 5 Barcelona Clinic Liver Cancer (BCLC) stage B patients received four intradermal DC vaccinations with a dose of 5 × 10^7^ cells at 2-week intervals after TACE; in group II, 5 BCLC stage B patients received TACE alone as a control group for group I; in group III, 5 BCLC stage D patients received four intradermal DC vaccinations with a dose of 5 × 10^7^ cells at 2-week intervals; and in group IV, 5 BCLC stage D patients received supportive treatment as a control group for group III. The results showed that DC vaccination was safe and well tolerated in all patients. In group I of patients, three (60%) developed a PR, and one (20%) achieved SD; however, no difference was observed in the clinical response between group I and II of patients. In group III of patients, one (20%) developed a PR, and two (40%) achieved SD; in contrast, in group IV of patients, none had a PR or SD. Both group I and group III of patients exhibited elevated frequency of peripheral CTLs and decreased serum levels of AFP after vaccination. Another completed phase I/II clinical trial (NCT03086564) evaluated the clinical results of an HBV-specific-antigen-peptides-co-pulsed and HepG2-cell-lysate-co-pulsed mature-DC vaccine combined with TACE in treating patients with unresectable HBV-related primary HCC. In another completed (JPRN-UMIN000020811) phase I clinical trial and ongoing phase I clinical trial (JPRN-jRCTc040190093), the clinical results of a peptides-pulsed mature-DC vaccine combined with RFA in treating patients with primary HCC were evaluated. However, there are no available results from the three trials mentioned. Another completed phase I/II clinical trial (JPRN-UMIN000010691) conducted by Matsui et al. evaluated the safety and efficacy of a HSP70 mRNA-transfected mature-DC vaccine combined with surgical resection in treating patients with resectable primary HCC [44]. A total of 45 patients were divided into two groups with no difference in baseline characteristics: in group I, 31 patients received three intradermal DC vaccinations with a dose of 2 × 10^6^ cells after surgical resection (first vaccination is 5 to 9 days after surgery, second vaccination is 5 to 10 weeks after surgery, and third vaccination is 9 to 16 weeks after surgery); and in group II, 14 patients received surgical resection alone as a control group. The results showed that DC vaccination was safe and well tolerated in all patients. Although there was no difference in the OS and disease-free survival (DFS) between the two groups of patients, in the subgroup of patients with HSP70-expressing HCC, the median OS and DFS times were significantly longer in group I than in group II (*p*-values = 0.003 and 0.090, respectively). Another completed phase I/II clinical trial conducted by Tada et al. evaluated the safety and efficacy of a mature-DC vaccine co-pulsed with AFP, MAGE-1, and GPC-3 proteins that was combined with TACE in treating five patients with primary HCC [45]. All the patients were vaccinated with 4 × 10^7^ of DCs subcutaneously four times, at 2-week intervals, 2 weeks after the first TACE. Four weeks following the first cycle of treatment, all the patients received two further vaccinations at 2-week intervals, 2 weeks after the second TACE. The results showed that DC vaccination was safe and well tolerated in all patients. Among all patients, one (20%) achieved SD, and five (100%) exhibited increased IFN-γ-producing CTL responses against AFP, MAGE-1, and/or GPC-3 antigens after vaccination. Another ongoing phase II clinical trial (KCT0000986) was further aimed at evaluating the clinical results of a mature-DC vaccine co-pulsed with AFP, MAGE-1, and GPC-3 proteins and given in combination with TACE in treating patients with unresectable primary HCC. Another completed phase II clinical trial (JPRN-UMIN000021545) evaluated the clinical results of a mature-DC vaccine co-pulsed with AFP, MAGE-1, and GPC-3 proteins cand given in combination with surgical resection in treating patients with unresectable primary HCC. However, there are no available results from the two trials mentioned. Another completed phase I/IIa (KCT0000427) clinical trial and completed phase II (KCT0000008) clinical trial, both conducted by Lee et al., evaluated the safety and efficacy of a mature DC vaccine co-pulsed with AFP, MAGE-1, and GPC-3 proteins and given in combination with surgical resection, RFA, PEI, or TACE in treating patients with primary HCC [46,47]. In the phase I/IIa trial, a total of 12 patients were vaccinated with 5 × 10^7^ of DCs, subcutaneously, six times (four vaccinations at 2-week intervals, followed by two vaccinations at 4-week intervals), 8 weeks after anticancer therapy. The results showed that DC vaccination was safe and well tolerated in all patients. Among all patients, nine (75%) did not develop tumor recurrence up to 24 weeks and displayed stronger IFN-γ-producing CTL responses against AFP, MAGE-1, and/or GPC-3 antigens than the others who developed recurrence after vaccination. The median TTP was significantly longer in the patients with vaccination than the patients without vaccination (36.6 months versus 11.8 months, *p*-value = 0.0031). In the phase II trial, a total of 156 patients were divided into two groups with no difference in baseline characteristics: in group I, 77 patients received six subcutaneous DC vaccinations with a dose of 3 × 10^7^ cells (four vaccinations at 2-week intervals, followed by two vaccinations at 4-week intervals) 4 weeks after anticancer therapy; and in group II, 79 patients received anticancer therapy alone as a control group. The results showed that DC vaccination was safe and well tolerated in all patients. In group I of patients, 63% exhibited enhanced IFN-γ-producing CTL responses against AFP, MAGE-1, and/or GPC-3 antigens after vaccination. Although there was no difference in the OS and recurrence-free survival (RFS) between the two groups of patients, in the subgroup of patients who were not treated with RFA, the RFS was significantly better in group I than in group II (*p*-values = 0.03). Another ongoing phase I clinical trial (NCT03674073) was aimed at evaluating the clinical results of an HCC-tumor-neoantigen-pulsed mature-DC vaccine combined with PMWA in treating patients with primary HCC. Another ongoing phase II clinical trial (NCT04912765) was aimed at evaluating the clinical results of an HCC tumor neoantigen-pulsed mature-DC vaccine combined with ICI nivolumab and surgical resection in treating patients with resectable primary or recurrent HCC. Another ongoing phase II clinical trial (NCT04317248) was aimed at evaluating the clinical results of a multiple HCC tumor antigens-pulsed mature-DC vaccine combined with surgical resection or TACE or the molecular targeted drugs sorafenib or lenvatinib in treating patients with HBV-related primary HCC. However, there are no available results from the three trials mentioned. Taken together, these clinical trials indicate that autologous-tumor-lysate-pulsed, allogeneic-tumor-cell-line-lysate-pulsed, or specific-tumor-antigen-pulsed DC vaccines are safe and effective as adjuvant therapy in combination with standard anticancer therapies for treating HCC patients; however, the efficacy still needs to be optimized in further studies.

### 3.3. Autologous-Tumor-Lysate-Pulsed or Specific-Tumor-Antigen-Pulsed DC Vaccines Together with Immune Effector Cells Combined with Anticancer Therapies

A completed phase II clinical trial conducted by Shimizu et al. evaluated the safety and efficacy of an autologous-HCC-tumor-lysate-pulsed mature-DC vaccine, together with CD3-activated T cells (CATs), that was combined with surgical resection in treating patients with primary HCC [48]. A total of 94 patients were divided into two groups, with no difference in baseline characteristics: in group I, 42 patients received both intradermal DC vaccination and intravenous CAT infusion three times, with doses of about 3.5 × 10^7^ and 2 × 10^9^ cells, respectively, within 2 months of surgical resection; and in group II, 52 patients received surgical resection alone as a control group. The results showed that DC vaccination together with CAT infusion were safe and well tolerated in all patients in group I. Group I of patients had significantly longer median OS (97.7 months versus 41.0 months, *p*-values = 0.029) and RFS (24.5 months versus 12.6 months, *p*-values = 0.011) times than the group II of patients. Another completed phase I clinical trial conducted by Zhou et al. evaluated the safety and feasibility of an autologous HCC-tumor-lysate-pulsed mature-DC vaccine together with immature DCs, cytokine-induced killer cells (CIKs, in vitro generated lymphocytes with a mixed NK and T cell-like phenotypes and functions), mature-DC-precision CTLs, and mature-DC-activated CIKs (DC-CIKs) combined with PMWA in treating 10 patients with HBV-related primary HCC [49]. All the patients received three courses of DC vaccination and immune effector cell infusion (first course was intratumoral immature DC infusion on the date of PMWA and intravenous CIK infusion on day 5 after PMWA; second course was intranodal DC vaccination and intratumoral CTL infusion on day 11 after PMWA; and third course was intranodal DC vaccination and intraperitoneal DC-CIK infusion on day 100 and intravenous CIK infusion on day 102 after PMWA). The results showed that DC vaccination in combination with immune effector cell infusion was safe and well tolerated in all patients. Among seven patients who did not receive antiviral therapy, four (57.1%) had decreased and two (28.6%) had undetectable serum levels of viral DNA after vaccination and infusion. Another ongoing phase II clinical trial (NCT01821482) was aimed at evaluating the clinical results of mature DC-CIKs combined with surgical resection or TACE in treating patients with primary HCC; however, the results are not available. Another completed clinical trial conducted by Zhou et al. evaluated the safety and efficacy of a mature-DC vaccine together with CIKs combined with the molecular targeted drug sorafenib in treating patients with advanced primary HCC [50]. A total of 71 patients were divided into two groups, with no difference in baseline characteristics: in group I, 35 patients received both the DC vaccination and CIK infusion after sorafenib treatment; and group II, 36 patients received sorafenib treatment alone as a control group. The results showed that DC vaccination, together with CIK infusion, was safe and well tolerated in all patients. After a 6-month follow-up period, in group I of patients, 4 (11.4%) achieved a CR, 14 (40%) displayed a PR, and 13 (37.1%) had SD. In contrast, in group II of patients, only one (2.8%) achieved a CR, five (13.9%) displayed a PR, and nine (25%) had SD. After a minimum follow-up period of 24 months, the median OS time was significantly longer in group I than in group II of patients (18.6 months versus 13.8 months, *p*-value < 0.05). Group I of patients exhibited significantly decreased serum levels of AFP after vaccination and infusion. Another ongoing phase I/II clinical trial (NCT02632188) was aimed at evaluating the clinical results of mature-DC-precision multiple-antigen CTLs combined with surgical resection in treating patients with primary HCC. Another ongoing phase I/II clinical trial (NCT02638857) was further aimed at evaluating the clinical results of mature-DC-precision multiple-antigen CTLs combined with TACE in treating patients with unresectable primary or recurrent HCC. However, there are no available results from the two trials mentioned. Another completed phase II clinical trial (NCT03067493) conducted by Peng et al. evaluated the safety and efficacy of a personalized HCC-tumor-neoantigen-pulsed mature-DC vaccine together with mature-DC-precision neoantigen CTLs combined with surgical resection or RFA in treating 10 patients with primary HCC [51]. All the patients received both subcutaneous DC vaccination (1.65 × 10^6^ to 1.88 × 10^7^ cells per dose) and intravenous CTLs infusion (0.56 × 10^6^ to 8.12 × 10^9^ cells per dose), with a median of 12 cycles and median of 16.6, 20.2 weeks after anticancer therapy. The results showed that DC vaccination, together with CTL infusion, was safe and well tolerated in all patients. Among all patients, five (50%) experienced no tumor recurrence for 2 years, and seven (70%) generated de novo multiclonal-neoantigen-specific CTL responses; the median DFS time was 18.3 months. Among the seven patients who generated immune responses, five (71.4%) did not develop tumor recurrence for 2 years; in contrast, all of the patients who did not generate immune responses developed tumor recurrence. The patients who generated immune responses exhibited a significantly better DFS than the patients who did not generate immune responses (*p*-value = 0.012). Overall, these clinical trials suggest that DC-based vaccines, together with immune effector cells as combination adjuvant therapy, are safe and effective in treating HCC patients who receive standard anticancer therapies; however, further studies are still needed to improve the efficacy.

## 4. Recent Preclinical Studies Regarding DC-Vaccine-Based Immunotherapy for HCC

Many promising strategies have been proposed and evaluated in recent in vitro and in vivo preclinical studies to optimize the efficacy of DC-vaccine-based immunotherapy for HCC (Table 3). A study performed by Jin et al. evaluated the efficacy of a mature-DC vaccine, which was stimulated with the sulfated glycoconjugate compound curdlan sulfate as a maturation reagent, in treating HCC [52]. The results showed that curdlan sulfate-stimulated DC vaccine exhibited comparable efficacy in suppressing tumor growth and enhanced efficacy in prolonging survival in an ectopic allograft immunocompromised BALB/c mouse model of the mouse HCC cell line H22 compared to the commonly used maturation reagent lipopolysaccharide (LPS)-stimulated DC vaccine. Another study performed by Chieochansin et al. evaluated the efficacy of mature-DC vaccines, which were pulsed with total cell lysate or total RNA prepared from a single human HCC cell line or combinations of two or three human HCC cell lines (Huh7, HepG2, and SNU449), in treating HCC [53]. The results showed that total the RNA-pulsed DC vaccines induced stronger IFN-γ-producing CTL-mediated cytotoxicity against each of the three human HCC cell lines in vitro than total-cell-lysate-pulsed DC vaccines. The cytotoxic activity of CTLs was further augmented when the DC vaccines were pulsed with total cell lysate or total RNA prepared from all three cell lines compared to either one or two cell lines. Another study performed by Pang et al. evaluated the efficacy of a mature-DC vaccine, which was fused with a flow cytometry-sorted cancer stem cell (CSC) marker CD90-positive irradiated HepG2 cell line, in treating HCC [54]. The results showed that CD90-positive irradiated HepG2 cell-fused DC vaccine induced stronger IFN-γ-producing CTL-mediated cytotoxicity against CD90-positive HepG2 cell line in vitro and exhibited better efficacy in suppressing tumor growth in an ectopic xenograft immunocompromised BALB/c mouse model of non-CD90-sorted HepG2 cell line than non-CD90-sorted irradiated HepG2 cell-fused DC vaccine. Another study performed by Zhou et al. evaluated the efficacy of a mature-DC vaccine, which was transfected with adenoviral vector DNA containing a gene encoding the HCC tumor marker aspartate-β-hydroxylase (AAH), in treating HCC [55]. The results showed that AAH DNA-transfected DC vaccine induced stronger IFN-γ-producing CTL-mediated cytotoxicity against human HCC cell line SMMC-7721 in vitro and exhibited better efficacy in suppressing tumor growth in an ectopic xenograft immunocompromised BALB/c mouse model than non-AAH DNA-transfected DC vaccine. Another study performed by Vogt et al. evaluated the efficacy of combination of two mature-DC vaccines, which were transfected with adenoviral vector DNA containing a gene encoding either the HCC tumor marker AFP or the immune co-stimulatory molecule CD40 ligand (CD40L), in treating HCC [56]. The results showed that combination of the two DC vaccines exhibited better efficacy in suppressing tumor growth and prolonging survival than either of the two DC vaccines alone in both ectopic and orthotopic allograft immunocompetent C3H/HeN mouse models of a stable AFP-expressing mouse HCC cell line, Hepa129. Another study performed by Xu et al. evaluated the efficacy of IFN-producing killer DCs (IKDCs, a subset of immune cells with certain phenotypes and functions of both DCs and NKs), which were transfected with lentiviral vector DNA containing a gene encoding the T-box family transcription factor T-bet for IFN-γ induction, in treating HCC [57]. The results showed that T-bet DNA-transfected IKDCs exhibited stronger cytotoxic activity against H22 cell line in vitro and better efficacy in suppressing tumor growth in an ectopic allograft immunocompetent C57BL/6 mouse model than non-T-bet DNA-transfected IKDCs. Another two studies performed by Teng et al. evaluated the efficacy of a mature-DC vaccine, which was pulsed with total cell lysate prepared from mouse HCC cell line Hep-55.1C, in combination with the ICIs that target either PD-1 or programmed death ligand 1 (PD-L1), in treating HCC [58,59]. The results showed that combination of the DC vaccine and ICIs induced stronger granzyme B-expressing CTL-mediated cytotoxicity against Hep-55.1C cell line in vitro and exhibited better efficacy in suppressing tumor growth and prolonging survival in an orthotopic allograft immunocompetent C57BL/6 mouse model than either the DC vaccine or ICIs alone. Another study performed by Wang et al. evaluated the efficacy of a DC-based nanoparticle vaccine, which was prepared by coating an acidic/photosensitive nanoparticle with the membrane of H22-cell-specific neoantigen-pulsed mature DCs, in combination with near-infrared (NIR) laser irradiation in treating HCC [60]. The results showed that the DC-based nanoparticle vaccine induced stronger IFN-γ-producing CTL-mediated cytotoxicity against the H22 cell line in vitro and exhibited better efficacy in suppressing tumor growth and prolonging survival in an ectopic allograft immunocompromised BALB/c mouse model than non-DC-based nanoparticle vaccine when combined with laser irradiation. Another study performed by Zuo et al. evaluated the efficacy of a DC-derived exosome vaccine, which was prepared by co-conjugating an immortalized DC-line-derived exosome with an AFP-derived epitope AFP_212_, an HCC tumor-targeting peptide P47, and a functional domain of high mobility group nucleosome-binding protein 1 (HMGN1), an immunoadjuvant capable of promoting DC recruitment and activation, in treating HCC [61]. The results showed that the DC-derived exosome vaccine exhibited efficient efficacy in suppressing tumor growth and prolonging survival in both ectopic and orthotopic allograft immunocompetent C57BL/6 mouse models of the mouse HCC cell line Hepa1-6. Altogether, these preclinical studies provide promising strategies to improve the therapeutic efficacy of DC-based vaccines for HCC, as such vaccines are worth conducting clinical trials for in order to evaluate their safety and efficacy in treating HCC patients.

**Table 3:** Summary of recent preclinical studies regarding DC-vaccine-based immunotherapy for HCC.

**Treatment**	**Experimental Model Applied**	**Experimental Results**	**Publication**
Curdlan sulfate–stimulated mature-DC vaccine	HCC mouse model	Comparable efficacy in suppressing tumor growth compared to LPS-stimulated DC vaccine Enhanced efficacy in prolonging survival compared to LPS-stimulated DC vaccine	Jin et al. [52]
Mature-DC vaccine co-pulsed with Huh7, HepG2, and SNU449 HCC cell lysate or RNA	HCC cell line	Better efficacy in inducing IFN-γ-producing CTL-mediated cytotoxicity by DC vaccine pulsed with RNA than cell lysate prepared from three cell lines than one or two cell lines	Chieochansin et al. [53]
CD90-positive irradiated-HepG2-HCC-cell-fused mature-DC vaccine	HCC cell line HCC mouse model	Better efficacy in inducing IFN-γ-producing CTL-mediated cytotoxicity than non-CD90-sorted irradiated HepG2 cell-fused DC vaccine Better efficacy in suppressing tumor growth than non-CD90-sorted irradiated HepG2 cell-fused DC vaccine	Pang et al. [54]
AAH-DNA-transfected mature-DC vaccine	HCC cell line HCC mouse model	Better efficacy in inducing IFN-γ-producing CTL-mediated cytotoxicity than non-AAH DNA-transfected DC vaccine Better efficacy in suppressing tumor growth than non-AAH DNA-transfected DC vaccine	Zhou et al. [55]
Combination of two mature-DC vaccines, one transfected with AFP DNA and the other transfected with CD40L DNA	HCC mouse model	Better efficacy in suppressing tumor growth than either of the two DC vaccines alone Better efficacy in prolonging survival than either of the two DC vaccines alone	Vogt et al. [56]
T-bet DNA-transfected IKDCs	HCC cell line HCC mouse model	Stronger cytotoxic activity than non-T-bet DNA-transfected IKDCs Better efficacy in suppressing tumor growth than non-T-bet DNA-transfected IKDCs	Xu et al. [57]
Hep-55.1C-HCC-cell-lysate-pulsed mature-DC vaccine combined with ICI against PD-1	HCC cell line HCC mouse model	Better efficacy in inducing granzyme B-expressing CTL-mediated cytotoxicity than either the DC vaccine or ICI alone Better efficacy in suppressing tumor growth than either the DC vaccine or ICI alone Better efficacy in prolonging survival than either the DC vaccine or ICI alone	Teng et al. [58]
Hep-55.1C-HCC-cell-lysate-pulsed mature-DC vaccine combined with ICI against PD-L1	HCC cell line HCC mouse model	Better efficacy in inducing granzyme B-expressing CTL-mediated cytotoxicity than either the DC vaccine or ICI alone Better efficacy in suppressing tumor growth than either the DC vaccine or ICI alone Better efficacy in prolonging survival than either the DC vaccine or ICI alone	Teng et al. [59]
H22-HCC-cell-specific neoantigen-pulsed mature-DC-membrane-coated acidic/photosensitive nanoparticle vaccine combined with NIR laser irradiation	HCC cell line HCC mouse model	Better efficacy in inducing IFN-γ-producing CTL-mediated cytotoxicity than non-DC-based nanoparticle vaccine combined with laser irradiation Better efficacy in suppressing tumor growth than non-DC-based nanoparticle vaccine combined with laser irradiation Better efficacy in prolonging survival than non-DC-based nanoparticle vaccine combined with laser irradiation	Wang et al. [60]
DC-derived exosome vaccine co-conjugated with AFP epitope AFP_212_, HCC tumor-targeting peptide, and HMGN1 functional domain	HCC mouse model	Efficient efficacy in suppressing tumor growth Efficient efficacy in prolonging survival	Zuo et al. [61]

Abbreviations: DC, dendritic cell; HCC, hepatocellular carcinoma; LPS, lipopolysaccharide; IFN-γ, interferon-gamma; CTL, cytotoxic T lymphocyte; AAH, aspartate-β-hydroxylase; AFP, alpha-fetoprotein; CD40L, CD40 ligand; IKDC, interferon-producing killer dendritic cell; ICI, immune checkpoint inhibitor; PD-1, programmed death 1; PD-L1, programmed death ligand 1; NIR, near-infrared; HMGN1, high-mobility-group nucleosome-binding protein 1.

## 5. Conclusions

**Figure 1:**
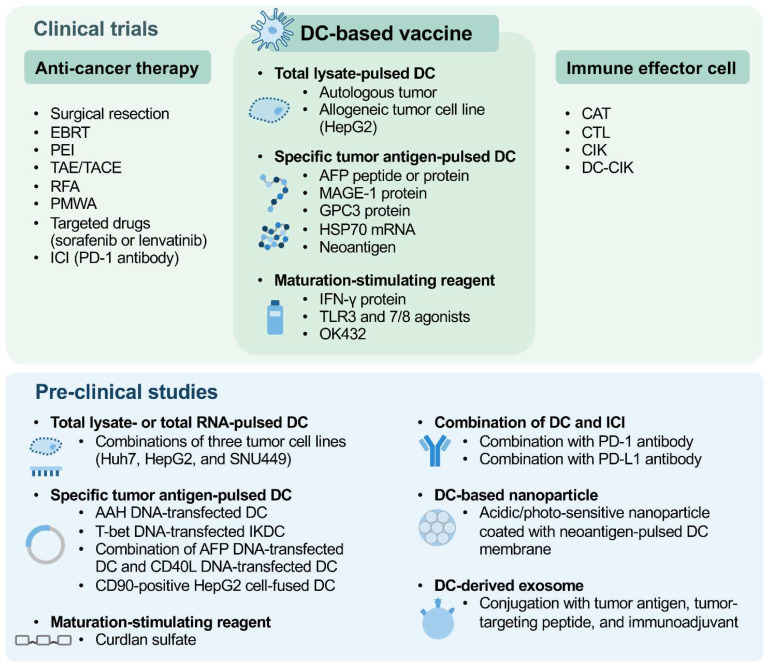
Schematic summary of clinical and preclinical strategies of DC-vaccine-based immunotherapy for HCC.

This review summarizes the evidence for various strategies evaluated in clinical trials and recent preclinical studies for the development of DC-vaccine-based immunotherapy for HCC (Figure 1), highlighting that a DC vaccine, whether alone or in combination with anticancer therapies and/or immune effector cells, holds great promise as a personalized therapeutic approach for treating patients with HCC. Some strategies focus on the optimization of DC vaccine efficacy, including the choice of tumor antigens pulsing with DCs and the discovery of DC maturation-stimulating reagents; some strategies focus on the combination of DC vaccines, either with or without immune effector cells, and current anticancer therapies; and others focus on the application of nanotechnology in DC vaccines or DC-derived exosomes. In addition, different injection routes and treatment regimens of DC vaccines are applied and evaluated in clinical trials. All of these parameters are critical in determining the therapeutic efficacy of DC-vaccine-based immunotherapy. Moreover, considering that HCC exhibits a high degree of heterogeneity in both the genomic landscape and immune microenvironment [62], it is therefore also important to identify biomarkers of treatment response for selecting the best treatment strategies for patients.

DC-based vaccines, which are prepared by different tumor-antigen-pulsing strategies or maturation-stimulating reagents, either alone or in combination with various anticancer therapies and/or immune effector cells for treating HCC patients, have been evaluated in clinical trials. Many promising strategies regarding tumor antigen pulsing, maturation stimulation, ICI combination, DC-based nanoparticles, and DC-derived exosomes have also been evaluated in preclinical studies to optimize the anti-HCC efficacy of DC vaccine-based immunotherapy.

## Abbreviations

DC, dendritic cell; IFN-γ, interferon-gamma; MAGE-1, melanoma-associated antigen 1; GPC-3, glypican-3; HSP70, heat-shock protein 70; mRNA, messenger RNA; TLR, Toll-like receptor; EBRT, external beam radiation therapy; PEI, percutaneous ethanol injection; TAE, transarterial embolization; TACE, transarterial chemoembolization; RFA, radiofrequency ablation; PMWA, percutaneous microwave ablation; ICI, immune checkpoint inhibitor; PD-1, programmed death 1; CAT, CD3-activated T cell; CTL, cytotoxic T lymphocyte; CIK, cytokine-induced killer cell; DC-CIK, dendritic-cell-activated cytokine-induced killer cell; AAH, aspartate-β-hydroxylase; CD40L, CD40 ligand; PD-L1, programmed death ligand 1.
